# Confirmatory factor analysis and measurement invariance of the Depression, Anxiety, and Stress Scale (DASS-21) among Pakistani young adults

**DOI:** 10.1192/j.eurpsy.2024.1081

**Published:** 2024-08-27

**Authors:** S. M. A. Kazmi, C. A. Lewis

**Affiliations:** ^1^Higher Education Department, Punjab, Pakistan; ^2^Psychology, Leeds Trinity University, Leeds, United Kingdom

## Abstract

**Introduction:**

The Depression, Anxiety and Stress Scale (DASS-21) is recognized as being a widely used measure for the assessment of negative emotional states. While the DASS-21 has been widely used for assessing mental health in Pakistan, limited research has been done regarding its factor structure and measurement invariance.

**Objectives:**

To assess the factor structure and measurement invariance of the DASS-21 among young adults in Pakistan.

**Methods:**

A large sample of 1361 Pakistani young adults had completed the scale during the current study comprising 666 males and 695 females with a mean age of 24.51 years.

**Results:**

Excellent internal consistency reliability was found for the overall DASS-21 and its three subscales (depression, anxiety and stress) ranging from *r* = .86 to .71 (Cronbach alpha). Moreover, the three subscales were strongly and significantly associated with one another. Additionally, the results showed a good fit of the three-factor model and the one-factor model of the DASS-21 aimed at assessing gender psychological distress. Strong measurement invariance was found regarding gender therefore showing that the DASS-21 is understood and interpreted similarly by males and females. However, little evidence was found regarding the three subscales (depression, anxiety and stress) for the measurement of three distinct constructs.

**Conclusions:**

These findings confirm the utility of the DASS-21 for measuring mental health in Pakistan among young adults.

**Disclosure of Interest:**

None Declared

